# Heavy Metal Resistance Determinants of the Foodborne Pathogen *Listeria monocytogenes*

**DOI:** 10.3390/genes10010011

**Published:** 2018-12-24

**Authors:** Cameron Parsons, Sangmi Lee, Sophia Kathariou

**Affiliations:** 1 Department of Food, Bioprocessing and Nutrition Sciences, North Carolina State University, Raleigh, NC 27695-7624, USA; skathar@ncsu.edu; 2 Seoul National University, Seoul 08826, Korea; slee19@ncsu.edu

**Keywords:** *Listeria monocytogenes*, heavy metal resistance, mobile genetic element, cadmium, arsenic

## Abstract

*Listeria monocytogenes* is ubiquitous in the environment and causes the disease listeriosis. Metal homeostasis is one of the key processes utilized by *L. monocytogenes* in its role as either a saprophyte or pathogen. In the environment, as well as within an animal host, *L. monocytogenes* needs to both acquire essential metals and mitigate toxic levels of metals. While the mechanisms associated with acquisition and detoxification of essential metals such as copper, iron, and zinc have been extensively studied and recently reviewed, a review of the mechanisms associated with non-essential heavy metals such as arsenic and cadmium is lacking. Resistance to both cadmium and arsenic is frequently encountered in *L. monocytogenes*, including isolates from human listeriosis. In addition, a growing body of work indicates the association of these determinants with other cellular functions such as virulence, suggesting the importance of further study in this area.

## 1. Introduction

*Listeria monocytogenes* is a Gram-positive facultative intracellular pathogen and the causative agent of the disease listeriosis. In healthy individuals, listeriosis can manifest as gastroenteritis; however, in at-risk individuals such as the elderly, pregnant women, or immunocompromised patients, listeriosis can result in severe symptoms, including septicemia, meningitis, stillbirths and even death [1,2,3]. Listeriosis is responsible for approximately 1455 hospitalizations and 255 deaths in the United States annually [4]. *L. monocytogenes* is found ubiquitously in the environment, is capable of growing in the cold, and can persistently colonize food production facilities [2,5]. This, along with the severe outcomes and life-threatening potential of listeriosis, makes *L. monocytogenes* a major cause for food safety and public health concern.

*L. monocytogenes* is well-adapted to survive both in the environment as well as within the body of humans and other animals [6,7]. One of the key adaptations for these dual survival modalities is metal homeostasis. Certain metals such as copper, zinc, and iron are required for essential cellular functions but become toxic at higher concentrations. In contrast, metals such as arsenic and cadmium appear to serve no cellular function and are considered toxic at any concentration [8]. In the environment, metals are typically found at low levels, but their concentrations can increase due to various anthropogenic interventions, including industrial pollution or agricultural practices [9,10]. In an animal host, metal concentrations are dependent on various factors, such as diet and tissue type [11,12,13]. The immune system can utilize metals in response to pathogens, either by restricting metal availability or by accumulating metals, to exert toxic effects on pathogens in the course of an infection [14,15,16]. For these reasons, the ability to import or export metals as needed is essential for *L. monocytogenes* to survive in its diverse environmental niches. Here we update the information currently available for functions related to the essential metals copper, iron, and zinc in *L. monocytogenes*, while providing the first comprehensive review of the widely-distributed resistances to toxic heavy metals, specifically cadmium and arsenic.

## 2. Essential Yet Potentially Toxic Metals

Metals such as copper, iron, and zinc are cofactors for essential enzymes, and insufficient amounts of these metals can result in cellular death [8]. However, at excessive concentrations these metals become toxic to the cells, disrupting membrane potential, interfering with enzyme function, and creating reactive oxygen species [8,17,18]. *L. monocytogenes* has several determinants to acquire these metals at highly regulated levels, and to expel, sequester, or convert and detoxify these metals when they are in excess [8,18,19,20]. Both conditions can occur in the animal host. Substantial work has been done to elucidate these processes for essential metals in *L. monocytogenes*, culminating in several reviews [8,18,19]. In relation to iron, recent findings have clarified the role of FrvA, which is implicated in haem toxicity and pathogenicity and is a high-affinity Fe(II)-exporting P-type ATPase with specificity for elemental iron [21,22]. Additionally, a recent study by Yousuf, Ahire, and Dicks elucidated the likely mechanism underlying copper toxicity in *L. monocytogenes* in which copper disrupts the cell membrane through lipid peroxidation and protein oxidation, as shown in other organisms as well [23,24,25]. Additionally of note from this study was the finding that *L. monocytogenes* had the most pronounced resistance to copper of all the Gram-positive organisms tested (*L. monocytogenes*, *Streptococcus* spp., *Enterococcus* spp. and *Bacillus cereus*), which was considered by the authors to be worthy of further investigation [25]. Recent work identified a dual role for the penicillin-binding protein encoded by *pbp4* (*lmo 2229* homolog), both in tolerance of *L. monocytogenes* to β-lactam antibiotics and in copper homeostasis [26].

## 3. Cadmium and Arsenic: Non-Essential Toxic Metals

In contrast to multiple reviews of *L. monocytogenes* determinants mediating homeostasis for essential metals, no comprehensive reviews are available on this pathogen’s resistance to non-essential toxic metals, such as arsenic and cadmium, even though resistance to these agents has been one of the earliest-documented phenotypes of *L. monocytogenes* [27,28]. Such resistance was encountered frequently enough to be utilized as a subtyping tool before the advent of higher-resolution techniques such as ribotyping, pulsed-field gel electrophoresis and multilocus sequencing, and was often associated with epidemic-associated clones [29,30]. Determinants mediating resistance to these heavy metals are widely distributed within *L. monocytogenes*, both on the chromosome and on plasmids [31,32,33,34,35,36]; all such determinants described here are summarized in Table 1.

## 4. Arsenic Resistance

Arsenic resistance has been primarily associated with serotype 4b, which is over-represented among clinical isolates in comparison to those from foods and food processing environments [29,39,40]. Further studies on the distribution of arsenic-resistant isolates within serotype 4b revealed that arsenic resistance was most frequently encountered among clones associated with outbreaks [30,36,39]. In particular, clonal complex (CC) 2 (formerly epidemic clone (EC) Ia) displayed the highest prevalence of arsenic-resistant isolates, and CC1 (previously ECI) was the second highest in the percentage of arsenic-resistant isolates, with resistance also encountered in several other clones, including the hypervirulent serotype 4b clone CC4 and isolates of CC315 and CC9 [30,36,39]. Interestingly, however, no CC6 (former ECII) isolates were found to be resistant to arsenic [30,36,39].

Albeit infrequent, arsenic resistance can also be found in serotypes 1/2a, 1/2b and 1/2c [39]. In serotype 1/2a, for instance, approximately 2% of the isolates tested were resistant to arsenic [39]. Interestingly, non-pathogenic *Listeria* species seem to largely lack arsenic resistance, with one of the exceptions being the reference strain *Listeria innocua* CLIP 11262, which harbors arsenic resistance genes on its plasmid pLI100 [31]. These findings suggest that arsenic resistance is primarily encountered in *L. monocytogenes*, especially in serotype 4b.

Analysis of strains that were persistently isolated from a rabbit meat processing facility in Italy revealed that approximately 90% of the isolates of clone CC14 (serotype 1/2a) exhibited arsenic resistance [41]. The extent to which arsenic resistance may contribute to persistence of this or other *L. monocytogenes* clones in food-processing facilities remains to be elucidated.

## 5. Arsenic Resistance Determinants

Typically, arsenic resistance cassettes are comprised of three (*arsRBC*) to five (*arsRDABC*) genes that are transcribed into a single polycistronic mRNA [37,42,43]. The genes *arsA* and *arsB* encode an ATPase and a membrane transporter, respectively, which form an ATP-dependent anion pump that exports arsenite from the cells [37,44]. The *arsA* gene product can function independently as a passive transporter of arsenite [37,42]. The *arsC* gene encodes a reductase that performs the conversion of arsenate to arsenite, which is then extruded by ArsA or the ArsA/ArsB complex [37,42,45,46]. Thus, deletion of *arsC* impairs resistance to arsenate but does not influence resistance to arsenite [45]. The *arsA*, *arsB*, and *arsC* genes are regulated by two regulatory proteins encoded by *arsR* and *arsD* [37,42,45,47]. The *arsR* gene product is a repressor that binds to the operator of the *ars* cassette in the absence of the inducer (arsenate and arsenite) but dissociates from the operator upon interaction with the inducer [37,45]. In other words, ArsR determines the basal expression level of the arsenic resistance cassette [37,45]. Meanwhile, the *arsD* gene product is not affected by the inducer and controls the maximal level of the *ars* operon, preventing the deleterious effects of *arsB* overexpression, such as hypersensitivity to arsenite [37,42,47].

Whole genome sequencing of *L. monocytogenes* has revealed three operons putatively associated with arsenic detoxification in *Listeria* spp. [31,34,35]. The first putative arsenic resistance operon (*arsR1D2R2A2B1B2*) was reported on plasmid pLI100, which is harbored in *L. innocua* CLIP 11262 [31,34]. As indicated above, no plasmid-borne arsenic resistance determinants have been reported in *L. monocytogenes*, which is consistent with earlier findings that arsenic resistance is chromosomally mediated in this species [29]. The other two putative arsenic resistance determinants are both located on the chromosome and were identified only in *L. monocytogenes*, each with a tendency to contribute to arsenic resistance in different serotypes. The first consists of the arsenic resistance cassette identified in pLI100 (*arsR1D2R2A2B1B2*) and two additional upstream genes, *arsD1* and *arsA1* [48]. The second consists of the arsenic resistance cassette *arsCBADR* harbored on a Tn*554*-like element [35].

The *arsR1D2R2A2B1B2* cassette, together with the upstream genes *arsD1* and *arsA1*, were initially identified on a 35-kb chromosomal island, termed *Listeria* genomic island 2 (LGI2), harbored by the CC2 strain Scott A [48]. Downstream of the arsenic resistance cassette, LGI2 also harbored the novel cadmium resistance determinant *cadA4* [35,36,48,49]. Therefore, all tested serotype 4b arsenic-resistant isolates were also resistant to cadmium [30,36].

Further studies using the LGI2 genes as genetic markers and whole genome sequence analysis showed that LGI2 was present in all tested arsenic-resistant isolates of serotype 4b, including isolates belonging to clones CC1 and CC2, and the hypervirulent clone CC4 [36,39,50]. Interestingly, the entire island was markedly diversified in a majority of arsenic-resistant CC1 strains; this diversified derivative was termed LGI2-1 and was inserted at the same chromosomal locus in all CC1 isolates that harbored it [36,39,50,51]. These findings suggest that arsenic resistance of serotype 4b can be attributable to arsenic resistance genes harbored on LGI2. Furthermore, regardless of the diversification, all serotype 4b arsenic-resistant isolates harboring LGI2 displayed tolerance to higher concentrations of arsenic (arsenite minimum inhibitory concentration (MIC) of 1.250 to 2.500 µg/mL) compared with susceptible strains (arsenite MIC of 250 to 500 µg/mL) [36,39]. However, direct experimental evidence is still warranted to explicitly demonstrate the involvement of arsenic resistance genes on LGI2 in arsenic detoxification.

LGI2 genes were rarely encountered among arsenic-resistant isolates that belong to serotypes other than 4b [39]. Even if strains were positive for LGI2 genes, both PCR typing and whole genome sequence analysis suggested sequence divergence from either LGI2 or LGI2-1, except in the case of a serotype 1/2a belonging to CC14, which harbored LGI2 that was highly conserved with that in the serotype 4b strain Scott A [39,41]. As mentioned earlier, CC14 isolates harboring this highly-conserved LGI2 were also found to be persistent in a rabbit meat processing plant [40].

While the genetic content of LGI2 is highly conserved in *L. monocytogenes*, genome analysis of arsenic-resistant isolates harboring LGI2 revealed this island to be inserted in at least eight different locations, primarily within open reading frames [39]. The GC content of LGI2 is lower than average (34% versus the *L. monocytogenes* average of 38%), and LGI2 also harbors a putative phage integrase gene. Such findings suggest that LGI2 was acquired via horizontal gene transfer from other bacterial genomes [36,39]. However, likely donors for LGI2 or LGI2-1 in *L. monocytogenes* remain unidentified.

The chromosomally-encoded arsenic resistance cassette (*arsCBADR*) that is associated with a Tn*554*-like transposon was first identified via whole genome sequencing of the serotype 1/2c strain SLCC 2372 [35]. When *arsA* associated with this Tn*554*-like element was used as a genetic marker, it was exclusively found among arsenic-resistant isolates of serotypes other than 4b, and approximately 90% of these isolates were negative for any LGI2-associated arsenic resistance genes, while positive for *arsA* harbored on the Tn*554*-like transposon [39]. These observations suggest that, in contrast to LGI2 which is predominantly found among serotype 4b isolates, the arsenic resistance cassette harbored on the Tn*554*-like transposon is responsible for arsenic resistance of strains of *L. monocytogenes* of other serotypes. Further experimental and *in-silico* evidence can deepen our understanding of the evolution and function of arsenic resistance associated with the Tn*554*-like element.

## 6. Cadmium Resistance

Several studies examined prevalence of cadmium resistance in strains isolated from food and food processing facilities in different regions [52,53,54,55]. Prevalence ranged from 50 to 66%, suggesting that cadmium resistance is globally widespread and highly prevalent in food-associated isolates. One study also noted that isolates repeatedly isolated from milk and dairy foods in Northern Ireland were more likely to be cadmium-resistant than those that were only sporadically recovered [56], suggesting that food or food processing facilities may provide unique pressures that select for cadmium resistance in *L. monocytogenes*.

In contrast to arsenic resistance often associated with serotype 4b, cadmium resistance was frequently encountered among isolates of serotype 1/2a, which are over-represented among food isolates compared with those of clinical origin [29,40]. In congruence with this association, cadmium resistance was generally much more prevalent than arsenic resistance among *L. monocytogenes* from foods and food processing environments [29,52,53,54,55]. Even in serotype 4b, the prevalence of cadmium-resistant isolates surpassed that of arsenic-resistant isolates; however, approximately 50% of serotype 4b cadmium-resistant isolates were also resistant to arsenic due to LGI2, as discussed above. While genes putatively associated with both cadmium and arsenic detoxification are co-localized on LGI2, multiple other cadmium resistance determinants have been identified in *L. monocytogenes* and are harbored both chromosomally and on plasmids, as will be discussed in the following section.

## 7. Plasmid-Associated Cadmium Resistance Determinants

In a survey of *L. monocytogenes* plasmids from strains of diverse origins (food, environmental, and clinical), an estimated 28% of the isolates were found to harbor plasmids, and most (95%) of these plasmid-harboring strains were found to be cadmium-resistant [28]. This was consistent with a later survey of plasmids in the genus *Listeria* that spanned multiple species, serogroups, and origins which found that, besides the origin of replication, the most common plasmid-borne elements were cadmium resistance cassettes encountered in all plasmids that were analyzed [34]. The cadmium resistance cassettes on the plasmids encoded a cadmium efflux P-type ATPase (*cadA*) and its putative repressor *cadC*. Multiple *cadA* determinants have been identified in *L. monocytogenes* and have been serially numbered, e.g., *cadA1*, *cadA2*, *cadA3*, etc., in the order in which they were discovered. Many *cadA*-harboring plasmids also harbored putative copper-resistance determinants [34]. Intriguingly, plasmid-encoded *cadA* in conjunction with a cassette of genes for arsenic detoxification has only been encountered once, in the aforementioned pLI100 of *L. innocua* CLIP 11262 [31,35]. The evolutionary and ecological mechanisms mediating the scarcity of pLI100-like plasmids with genes for both cadmium and arsenic resistance remain unclear.

**Figure 1:**
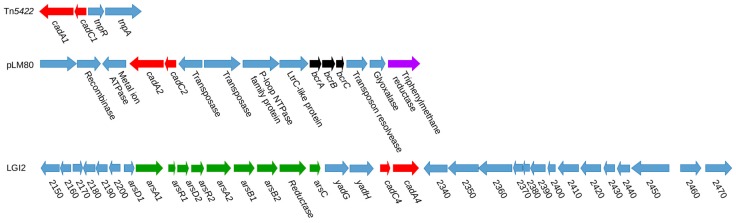
Resistance gene distribution across mobile genetic elements for *cadA1*, *cadA2*, and *cadA4*. *Cad* family members, benzalkonium chloride resistance determinants, toxic triphenylmethane dye resistance determinants, and putative arsenic detoxification determinants are in red, black, purple, and green, respectively.

The first *cadA* determinant to be identified in *L. monocytogenes* was *cadA1*, which was genetically similar to the *cadA* characterized in *Staphylococcus aureus* [38]. While the *cadA1* in *S. aureus* conferred resistance to both cadmium and zinc, the plasmid-harbored *cadA1* in *L. monocytogenes* was specific to cadmium [38]. *CadA1* was harbored on the mobile genetic element Tn*5422* [57]. Interestingly, Tn*5422* was never detected chromosomally but appeared to integrate extensively into plasmids, leading Lebrun et al. to speculate that this element was responsible for much of the size variation of plasmids in *L. monocytogenes* [38]. Tn*5422*-associated *cadA1* has been subsequently identified on numerous other plasmids of *L. monocytogenes* (Figure 1) [57,58].

A second putative *cadA* (*cadA2*) was first identified on the large plasmid pLI100 of *L*. *innocua* CLIP 11262, followed by its discovery on the approximately 80 kb plasmid pLM80 of *L. monocytogenes* H7858, a strain implicated in a large, multistate outbreak in the U.S. in 1998–1999, which involved contaminated hotdogs [31,33] (Figure 1). The latter plasmid was later experimentally confirmed to confer not only cadmium resistance but also resistance to the quaternary ammonium compound (QAC) benzalkonium chloride, via the *bcrABC* efflux cassette that mediated enhanced tolerance to benzalkonium chloride and other QACs [32,59]. In addition, pLM80 conferred resistance to toxic triphenylmethane dyes such as crystal violet and malachite green via *tmr*, a determinant that detoxifies these dyes and appears to have been acquired from Gram-negative bacteria [32,60] (Figure 1).

Plasmids harboring *cadA1* and *cadA2* have been observed in both *L. monocytogenes* and other, non-pathogenic *Listeria* spp. [34,61]. Several studies have suggested that in *L. monocytogenes*, *cadA1* is more common than *cadA2* [53,54,62]. Data also suggest that *cadA1* may have been predominant earlier in *L. monocytogenes* [28,38,57], since *cadA2* was not identified until the characterization of strains implicated in the 1998–1999 hot dog outbreak [32,33]. It is thought-provoking that pLM80-like plasmids were not identified previously in *L. monocytogenes* and that *cadA2* determinants were not detected among the cadmium-resistant plasmid-harboring isolates in earlier studies [28,57]. It is tempting to speculate that the reasons are related to co-selection of *cadA2*-harboring plasmids for resistance to QACs, which were not recognized as disinfectants until 1934 [63] and which have been routinely and extensively employed for sanitation of food processing facilities only in the past few decades. A survey of *L. monocytogenes* isolates of primarily serotype 1/2a or 1/2b from turkey processing plants in the U.S. revealed that cadmium-resistant isolates that also exhibited enhanced QAC tolerance were more likely to harbor *cadA2,* either alone or together with *cadA1*, than isolates that were cadmium resistant but without enhanced tolerance to QACs [62].

Strains harboring both *cadA1* and *cadA2* were encountered in some surveys [54,62], while other studies reported little or no co-occurrence of *cadA1* and *cadA2* in the same strains [36,53]. Such findings suggest that certain environments may be more conducive to the co-occurrence of these two *cadA* determinants. Analysis of *Listeria* plasmid sequences failed to reveal plasmids that harbor both *cadA1* and *cadA2* [34], suggesting that strains positive for both *cadA1* and *cadA2* harbored these determinants on different plasmids.

Overall, *cadA1* and *cadA2* were far more prevalent in serotype 1/2a and 1/2b strains from food and food processing plants in comparison to serotype 4b [53,62]. This could potentially be explained by the overall greater prevalence of plasmids in serogroup 1 than in serogroup 4 [34]. Strains that harbored only *cadA1* were significantly more likely to be serotype 1/2a, while those harboring only *cadA2* were significantly more likely to be serotype 1/2b [62]. An unexpected finding has been the lack of detection of *cadA1* among serotype 4b clinical isolates belonging to the major clones CC2 and CC6, while *cadA2* was not detected in another leading clone, CC1, suggesting proclivity of different clonal groups for specific cadmium resistance determinants [36,39]. The underlying reasons for differential prevalence of different *cadA* determinants in various serotypes and clones of *L. monocytogenes* are worthy of further investigation and may reflect differences in their ecology, including the microbial community that may include donors for the plasmids, and the accompanying selective pressures.

## 8. Chromosomal Cadmium Resistance Determinants

The proliferation of whole genome sequencing (WGS) data resulted in the discovery of several cadmium resistance determinants harbored chromosomally in *L. monocytogenes*. The first such determinant was *cadA3*, harbored on a mobile integrated conjugative element in strain EGDe [31]. While direct experimental evidence for its role in cadmium resistance is still lacking, the presence of this gene has been associated with tolerance to cadmium of >140 μg/mL [36]. Thus far, this determinant has been encountered infrequently, having been identified only in EGDe and a few additional strains [35,36,53]. A survey of 136 serotype 4b isolates from human sporadic listeriosis in the U.S. revealed 45 that were cadmium resistant, of which only one harbored *cadA3* [36].

Another chromosomal *cadA* family member (*cadA4*) was identified within the previously-discussed LGI2 in the chromosome of strain Scott A [35,36,48] (Figure 1). While *cadA1–3* are all associated with tolerance to cadmium of >140 μg/mL and can be detected through routine screening of the isolates on 70 μg/mL [29,52], *cadA4* only confers resistance to approximately 50 μg/mL of cadmium and thus can be detected by growth at 35 but not at 70 μg/mL [29,36,49,52,53]. Thus, resistance mediated by *cadA4* would have been undetected if isolates were screened at a level of 70 μg/mL as employed in studies prior to 2013 [28,29,52,53]. For this reason, the prevalence of cadmium resistance in *L. monocytogenes* may have been underestimated in earlier studies. Mechanisms underlying differences in resistance level between *cadA4* and *cadA1–A3* remain to be identified, but may reflect the divergent nature of the deduced *cadA4* product. Pairwise comparisons revealed that the *cadA4* amino acid sequence had approximately 36% identity to those encoded by *cadA1–A3*, while the latter exhibited approximately 70% identity to each other [36,49].

Due to its more recent characterization, *cadA4* has not been surveyed as extensively as *cadA1* and *cadA2*, and its distribution was mostly investigated in serotype 4b strains often associated with the arsenic resistance island LGI2 [30,36,39]. In a survey of 136 serotype 4b isolates from sporadic cases of human listeriosis in the United States discussed above, *cadA4* accounted for approximately 10% of the isolates and 29% of the cadmium-resistant isolates [36]. Interestingly, *cadA4* was always located on LGI2 downstream of arsenic resistance genes [39]. Even though *cadA4* has been primarily reported in serotype 4b, it has also been detected in a few strains of other serotypes (1/2a, 1/2b and 1/2c), including the persistent clone CC14 (serotype 1/2a), where *cadA4* was located on LGI2 downstream to arsenic resistance genes, as in serotype 4b [39,41]. To date, we lack reports of *cadA4*-harboring isolates of *Listeria* spp. other than *L. monocytogenes*.

**Figure 2:**
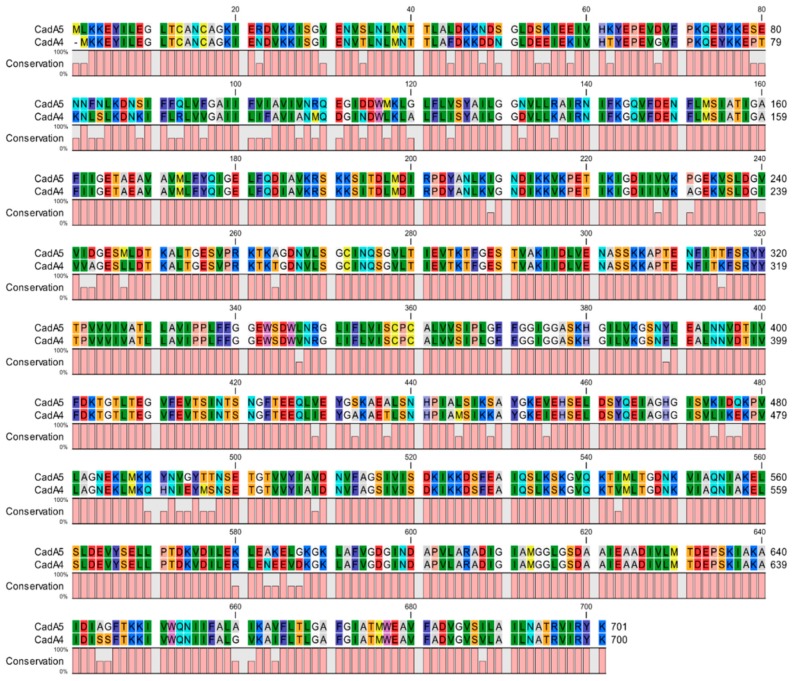
Amino acid alignment between *cadA4* in strain Scott A and its divergent counterpart (here labeled as *cadA5*) encountered in LGI2-1. Amino acid alignment was generated using CLC Genomics Workbench 11.0 [64].

As discussed above, a diversified LGI2 derivative (LGI2-1) was observed in a subset of arsenic-resistant serotype 4b CC1 isolates, and another derivative was also identified in a serotype 1/2c strain of CC9 [39]. The *cadA4* homolog in these divergent islands exhibited ~90% amino acid identity with the *cadA4* of Scott A (Figure 2). Together, LGI2-associated *cadA4* and its divergent homolog in LGI2-1 accounted for half of serotype 4b cadmium-resistant isolates in a previous study [30]. Further studies need to be conducted to investigate the prevalence and functional characteristics of this or additional *cadA4* homologs.

## 9. Impacts of Heavy Metal Resistance Determinants on other Adaptations, Including Virulence

There is a growing body of evidence that suggests links between heavy metal resistance and the ability of *L. monocytogenes* to cause disease. For instance, an investigation of the prevalence of arsenic and cadmium resistance among serotype 4b isolates from human listeriosis patients in the United States found a high prevalence of resistance and a strong association with clones repeatedly implicated in outbreaks [30]. More direct evidence supporting the involvement of cadmium resistance with pathogenicity has been provided in several studies. The first experimental evidence for the possible involvement of cadmium resistance determinants in virulence was obtained from *in-vivo* transcriptional analysis of *L. monocytogenes* from the livers of mice infected with strain EGDe, which harbors *cadA3* [65]. The putative repressor encoded by *cadC3* was markedly upregulated in the liver of the infected mice, and deletion of this gene resulted in a decrease in virulence when the bacteria were administered intravenously [65]. Similar findings were also reported by Pombinho et al. in 2017, who again identified *cadC3* as being essential for virulence of *L. monocytogenes* [66]. They found that, in addition to repressing *cadA3*, *cadC3* also repressed *ispB,* which is involved in initiating an immune response, thus helping *L. monocytogenes* avoid detection by the host immune system [66]. Interestingly, a transposon insertion mutant of *cadA4* [49] showed increased virulence in the *Galleria mellonella* model, suggesting an inverse relationship between *cadA4* and virulence [49]. This finding is consistent with the previous study, which demonstrated that the putative repressor *cadC3* was required for full virulence [65]. Taken together, the results of these studies suggest an association between cadmium resistance determinants and the ability of *L. monocytogenes* to cause disease. It is important to note, however, that these studies have focused on *cadC3* and *cadA4*. We currently lack information on the potential virulence or pathogenicity roles of the predominant cadmium resistance determinants *cadA1* or *cadA2*, and their cognate *cadC* repressors (*cadC1* and *cadC2*, respectively).

The cadmium and arsenic resistance genes of *L. monocytogenes* discussed here are accompanied by transcriptional regulators of the ArsR family of metal-associated transcriptional regulators [67]. In other bacterial systems, these regulators have been found to regulate expression of single genes in some circumstances, while mediating a global transcriptional response in others [67]. Most often, they regulate expression of genes directly involved in metal detoxification, but they can also impact expression of genes with a variety of other functions including oxidative stress tolerance, acid adaptation, respiration and ribosome biogenesis [67].

Members of the ArsR family were involved in the regulation of virulence-associated genes in various species. For instance, the PhoPR two-component regulatory system which is responsible for the regulation of virulence and persistence genes in *Mycobacterium* spp. was shown to be under the control of an ArsR transcriptional factor [68]. In *L. monocytogenes*, the aforementioned cadmium resistance regulator *cadC* belongs to the ArsR family and, as previously stated, *cadC3* was involved in virulence via its impacts on *ispB* [49,65,67].

In the case of the arsenic resistance genomic island LGI2, it is tempting to speculate that this element may have roles in virulence and pathogenicity, based on the fact that, as discussed above, LGI2 has been detected exclusively in *L. monocytogenes* and primarily in serotype 4b, which makes significant contributions to human listeriosis [56]. However, direct experimental evidence is needed to assess LGI2’s roles in virulence, e.g., by comparing virulence of isogenic strains with and without specific LGI2-associated genes. This was pursued with *cadA4* [49], but similar investigations with the arsenic resistance genes on LGI2 are lacking.

The potential contributions of heavy metal resistance to environmental persistence of *L. monocytogenes* remains to be elucidated. As discussed earlier, one study found that isolates repeatedly isolated from contaminated foods were more likely to be cadmium-resistant than those that were only sporadically encountered [56], and 74% or more of the serotype 1/2a and 1/2b isolates from turkey processing facilities were cadmium resistant, harboring *cadA1* and/or *cadA2* [52,62]. It is thus tempting to speculate that cadmium resistance via these determinants may enhance the capacity of the isolates to persist in the contaminated food or food processing environments, but the underlying mechanisms remain unknown.

In addition to their overt impacts on enhanced tolerance to heavy metals, heavy metal resistance genomic islands may indirectly influence environmental fitness or pathogenicity by promoting the horizontal transfer of accessory genes. In bacteria, metal resistance genes have been found to co-localize with antibiotic and other resistance genes on mobile genetic elements, such as plasmids, genomic islands, and transposons [69,70,71,72] (Figure 1). As discussed above, pLM80 and related plasmids harbor not only cadmium resistance genes but also genes mediating enhanced tolerance to QACs and toxic dyes [32,34,60]. Sequence data suggest that these elements have been introduced to *L. monocytogenes* from other species [48,60], and there is direct experimental evidence for the transfer of such elements between *L. welshimeri* or *L. innocua* and *L. monocytogenes* [61]. This creates the possibility that metal contamination and/or metal resistance genes could facilitate the acquisition and transfer of other resistance genes to *L. monocytogenes*, or from *L. monocytogenes* and other *Listeria* spp. to other bacterial agents of public health concern. It has also been shown that extremely low levels of metals can induce transcription of metal resistance genes and exert sufficient selective pressure to result in the retention of these elements [49,57,73]. These data suggest that in minute amounts such as might be encountered in the environment or in an animal host, heavy metals can potentially exert selective pressure, which in turn could direct the acquisition or transfer of mobile genetic elements that can impact the environmental or *in-vivo* fitness of *L. monocytogenes*.

## 10. Conclusions

Metals play a key role in the survival of *L. monocytogenes* both in the environment and in animal hosts [8]. Essential metals must be acquired, and toxic effects of excess metals must be mitigated. While the cellular functions of *L. monocytogenes* associated with essential metals have been extensively studied and reviewed, those involved with exclusively toxic metals such as cadmium and arsenic are poorly understood. The significance of these determinants is shown by their wide distribution within *L. monocytogenes,* as well as their association with food, food processing plants, clinical strains and clonal groups involved in outbreaks [36,53,54,55,62]. Evidence from other microorganisms suggests the involvement of metal resistance genes in a variety of functions beyond just metal detoxification [24,74]. Several studies discussed here would also suggest alternate and additional functions for these genes in *L. monocytogenes* [49,65]. Given their prevalence, potential involvement in selection and population dynamics, as well as their growing implication in important alternative cellular functions such as virulence, heavy metal resistance genes are an ideal candidate for further study.

## Figures and Tables

**Table 1:** Heavy metal resistance-associated determinants in *Listeria monocytogenes*.

Metal Resistance-Associated Determinant	Annotation
*arsA*	Arsenic efflux ATPase [37]
*arsB*	Membrane transporter [37]
*arsC*	Arsenate reductase [37]
*arsD*	Transcriptional regulator [37]
*arsR*	Transcriptional regulator [37]
*cadA* ^1^	Cadmium efflux ATPase [38]
*cadC* ^1^	Transcriptional regulator [38]

^1^ Cadmium resistance determinants can exhibit sequence divergence sufficient to be considered as different alleles of *cadA* and *cadC*. As discussed in this review, these have been designated with numbers, e.g., *cadA1 cadC1*, *cadA2 cadC2*, etc., based on the order in which they were identified or characterized.
